# Management of a gluteal region impalement injury caused by three reinforced aluminum bars: a case report

**DOI:** 10.1186/1752-1947-7-295

**Published:** 2013-12-31

**Authors:** Takashi Kanemura, Toru Hifumi, Ichiro Okada, Nobuaki Kiriu, Tomoko Ogasawara, Eiju Hasegawa, Hiroshi Kato, Yuichi Koido, Junichi Inoue

**Affiliations:** 1 National Disaster Medical Center, 3256 Midoricho, Tachikawa, Tokyo 190-0014, Japan; 2 Kagawa University Hospital, 1750-1 Ikenobe, Miki, Kita, Kagawa 761-0793, Japan; 3 Yamanashi Prefectural Central Hospital, 1-1-1 Fujimicho, Kofu, Yamanashi 400-8506, Japan

## Abstract

**Introduction:**

Impalement injuries with multiple objects are rare and their management is complex. Rapid confirmation of vascular injuries requiring urgent endovascular or surgical management and accurate location of multiple objects are essential for efficient preoperative management. We report the case of a patient with septic shock secondary to a perforated rectum caused by an impalement injury with three reinforced aluminum bars.

**Case presentation:**

A 58-year-old Asian man fell from the roof of a house and received gluteal impalement injuries from three reinforced aluminum bars. A physical examination showed paralysis of his left leg and no active bleeding from the insertion sites of the impaled objects. Multidetector computed tomography angiography confirmed the location of the aluminum bars, which had spared his small bowel, ureter and major vessels. No significant extravasation was observed. Two bars were successfully removed under general anesthesia in the lithotomy position. The third bar, which pierced his rectum, passed through the left side of his vertebrae and extended up to the superior side of his left kidney, was removed following a celiotomy. After removal of this bar, bleeding from the anterior side of the sacral bone was controlled by gauze packing. After surgery, our patient was admitted to our intensive care unit under endotracheal intubation and mechanical ventilation. Dopamine therapy was initiated, followed by direct hemoperfusion with polymyxin B-immobilized fiber (PMX-DHP) for septic shock secondary to a perforated rectum. This treatment was continued for two hours, resulting in stabilization of our patient’s hemodynamic condition. Daily peritoneal lavage was performed for several days, along with a colostomy. Although there were motor and sensory disturbances below the L3 level, there were no complications. On day 191 of admission, our patient was discharged with motor and sensory disturbances below the L3 level. He now uses a wheelchair and depends on assistance from others for daily activities.

**Conclusion:**

Preoperative multidetector computed tomography angiography confirmed the anatomic location of the aluminum bars and the absence of extravasation; these findings aided in treatment planning. Our patient was successfully managed by colostomy and aggressive surgical and critical care including direct hemoperfusion with polymyxin B-immobilized fiber, and developed no intra-abdominal infection or meningitis.

## Introduction

Impalement injuries with multiple objects are rare and their management is complex [1]. Careful planning for the removal of multiple objects is essential to decrease blood loss and preserve organ function; however, time is of the essence when evaluating and resuscitating such patients. Rapid confirmation of vascular injuries requiring urgent endovascular or surgical management and the accurate location of multiple objects are essential for efficient preoperative management [2-4]. The postoperative course in patients with injuries of this type is usually complicated, requiring aggressive critical care [5].

Here, we report the case of a 58-year-old Asian man who developed septic shock secondary to a perforated rectum caused by an impalement injury with three reinforced aluminum bars. He was successfully managed using preoperative multidetector computed tomographic angiography (MDCTA) and postoperative direct hemoperfusion with polymyxin B-immobilized fiber (PMX-DHP).

## Case presentation

**Figure 1:**
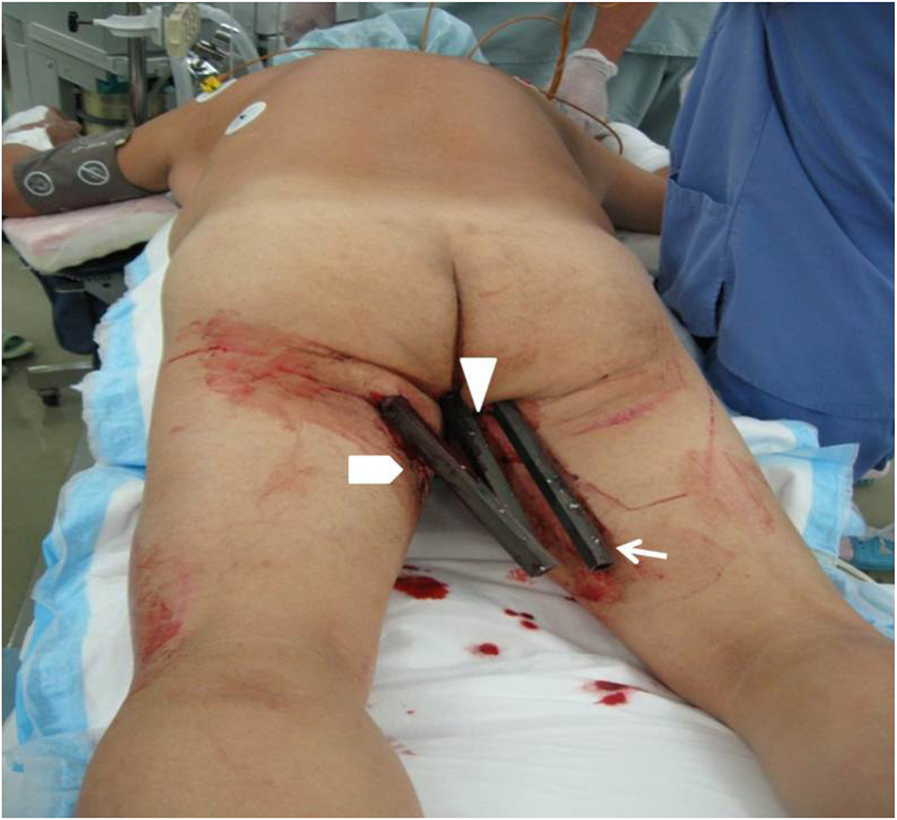
**Patient impaled with three steel bars:bar A (⌂; patient’s left side), bar B (∆; center) and bar C (↑; right).**

**Figure 2:**
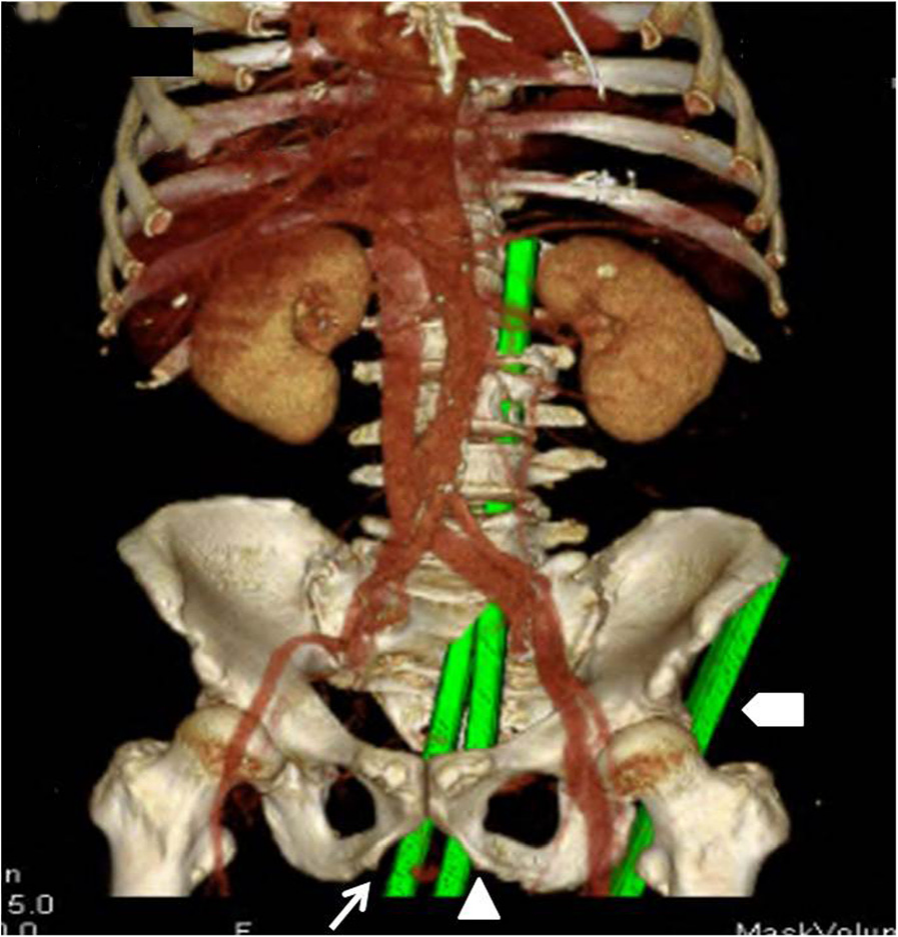
**Computed tomography reconstruction showing the location of the three steel bars: bar A (⌂; left), bar B (∆; center), and bar C (↑; right).** Bar A (left) pierced the left gluteal region and extended into the left anterior superior iliac spine. Bar B (center) pierced the right gluteal region, passed through the rectum, continued into the spinal canal from the sacral bone, and extended into the spinal canal at level L4. Bar C (right) pierced the perineal region, passed through the rectum, continued into the spinal canal from the sacral bone, exited the spinal canal at the left side of L3, and extended into the posterosuperior side of the left kidney.

**Figure 3:**
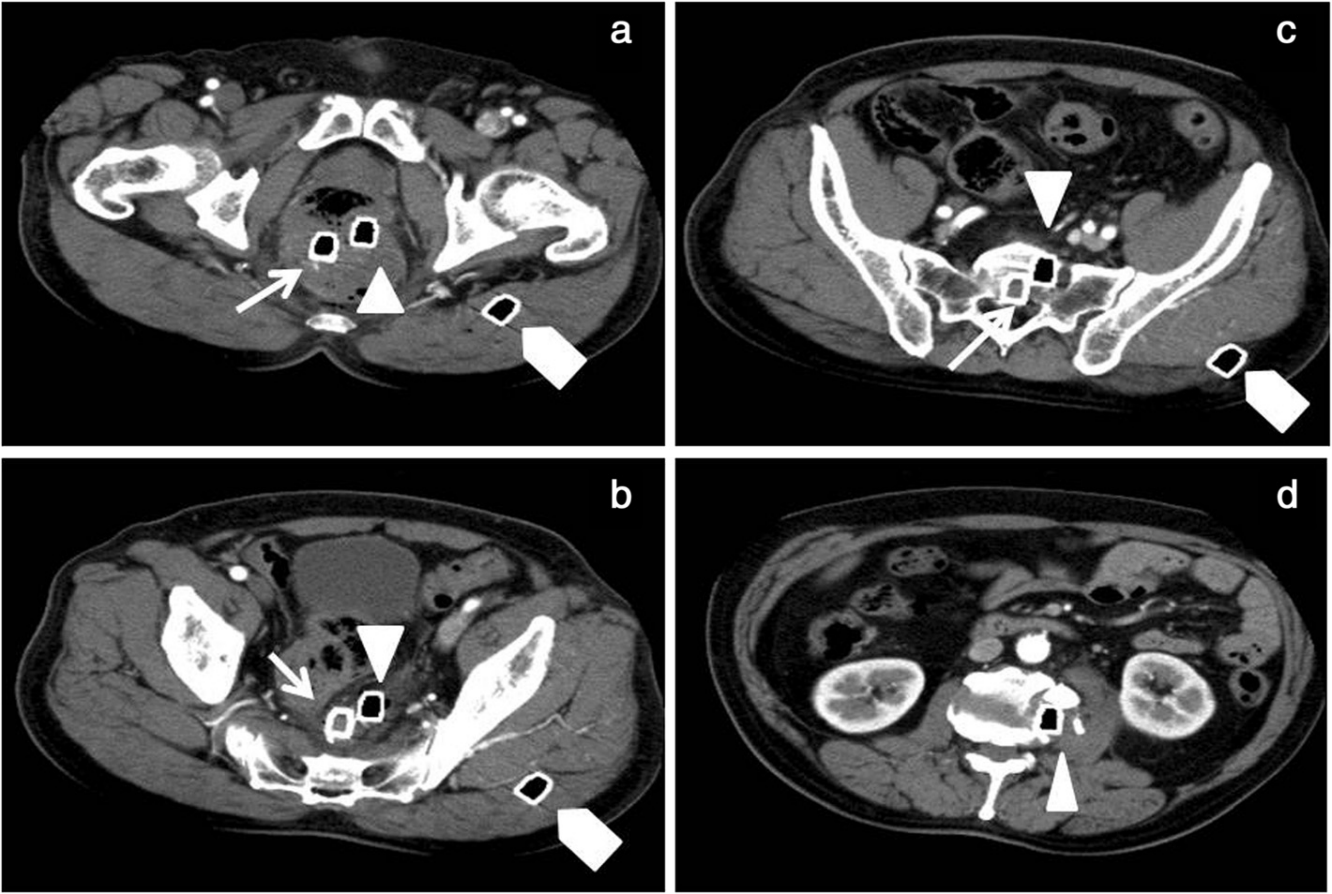
a-d. Abdominal computed tomography images (axial view) with three steel bars: bar A (⌂), bar B (∆) and bar C (↑).

A 58-year-old Asian man received gluteal impalement injuries from three reinforced aluminum bars measuring 2.0cm in diameter after falling from a roof. On presentation, he was fully conscious with normal vital signs. A physical examination revealed left leg paralysis and no active bleeding from the insertion sites of the aluminum bars (Figure 1). The location of the bars (A, B and C) was confirmed by MDCTA (Figures 2 and 3). Bar A (left) pierced his left gluteal region and extended into his left anterior superior iliac spine. Bar B (center) pierced his right gluteal region, passed through his rectum, continued into his spinal canal from the sacral bone, and extended into his spinal canal at the L4 level. Bar C (right) pierced his perineal region, passed through his rectum, continued into his spinal canal from the sacral bone, exited his spinal canal from the left side of L3, and extended into the posterosuperior side of his left kidney, sparing his small bowel, ureter and major vessels.

With our patient under general anesthesia and in the lithotomy position, bars A and B were gently pulled and successfully removed. Next, a celiotomy was performed to remove bar C. Following the removal of bar C, bleeding from the anterior side of his sacral bone was controlled by gauze packing. The perineal wound was debrided and presacral drainage was performed. The total volume of intraoperative bleeding was 4000mL, and 12 units of red cell concentrate and six units of fresh frozen plasma were transfused. Imipenem/cilastatin and clindamycin were preoperatively administered. After the surgery, our patient was admitted to our intensive care unit under endotracheal intubation and mechanical ventilation. Dopamine therapy was initiated, followed by PMX-DHP for the septic shock. He initially received 8μg/kg/min of dopamine, and was weaned off the catecholamine during the first two hours of PMX-DHP therapy. This resulted in stabilization of our patient’s hemodynamic condition after two hours. Daily peritoneal lavage was performed for several days, along with a colostomy. He was extubated on day 15 of admission and transferred to a general ward on day 16.

Although motor and sensory disturbances were observed below the L3 level, there were no complications, including meningitis or abscess formation. He developed a neurogenic bladder, which was managed by intermittent catheterization. On day 191 of admission, our patient was discharged with motor and sensory disturbances below the L3 level. At time of discharge, he required a wheelchair and depended on assistance from others for daily activities.

## Discussion

Preoperative management of multiple impalement injuries presents a challenge for emergency physicians because of the possible existence of multiple life-threatening injuries, and/or the unknown nature and extent of injury. Reports suggest that such patients should be rapidly assessed by targeted examination [6]. Radiographic or other time-consuming evaluations should not delay definitive treatment [6]; however, possible multisystem damage should be critically assessed [7]. In every patient with perineal injury, combined rectal and urinary tract injury should be ruled out [8].

MDCTA is the recommended primary imaging modality for the evaluation of trauma, especially for vascular injuries [9]. MDCTA enables whole body imaging in less than 30 seconds; this represents a major advantage during the evaluation of cases such as the one reported here. Advanced reconstructions facilitate the viewing of images with minimal interference from metallic objects [10]. In our patient, preoperative MDCTA confirmed that the bars had spared his small bowel, ureter and major vessels; therefore, we were able to plan a management strategy involving gentle manipulation and removal of two bars followed by surgical removal of the third. Minimal manipulation of the objects was mandatory to preserve any potential tamponade effects and control blood loss [11].

Our patient was managed by aggressive postoperative critical care, including PMX-DHP, which reportedly improves the outcome of colorectal perforation [12]. To the best of our knowledge, there are no reports on the efficacy of PMX-DHP for traumatic rectum perforation; however, PMX-DHP treatment has exerted beneficial effects on hemodynamics, pulmonary oxygenation and mortality in patients with septic shock [13,14]. Our patient’s hemodynamic status stabilized shortly after the initiation of PMX-DHP; therefore, we could concentrate our efforts on infection control.

## Conclusion

In patients with multiple gluteal impalement injuries, preoperative evaluation with MDCTA is warranted, and PMX-DHP is suggested as an aggressive, adjunct, critical care modality for treating anorectal injuries.

## Consent

Written informed consent was obtained from the patient for publication of this case report and accompanying images. A copy of the written consent is available for review by the Editor-in-Chief of this journal.

## Abbreviations

MDCTA: multidetector computed tomographic angiography; PMX-DHP: direct hemoperfusion with polymyxin B-immobilized fiber.

## Competing interests

The authors declare that they have no competing interests.

## Authors’ contributions

TK, TH, IO, NK, HK, YK and JI collected patient data and administered therapy. TH wrote the manuscript. TO, EH and JI revised and edited the manuscript. All authors read and approved the final manuscript.
